# Supplementary material for: Inequalities in care for the people with diabetes in Brazil: A nationwide study, 2019
Source: PLoS One. 2022 Jun 29;17(6):e0270027. doi: 10.1371/journal.pone.0270027 (PMC9242508; doi:10.1371/journal.pone.0270027)
Supplement: Attachment — Submitted filename: Response to Reviewers.odt [file pone.0270027.s001.odt]

August 20th, 2021 
To the Journal Plos One
Subject: answer to editors and reviewers

Dear PLOS ONE Editor 
We thank you for your careful review of the manuscript entitled “Inequalities in care for the people with diabetes in Brazil: a nationwide study, 2019”. We would like to point out that all requests have been met. Below are the details of each one and their respective answer.  
1) English language must be revised. 
Answer: the English language has been revised throughout the manuscript.
2) Methodology needs to be improved with a better explanation on the estimation of both indices: the slope index of inequality (SII) and the concentration index (CIX) and why they are appropriate for this specific study. 
Answer: in the penultimate paragraph of the methodology, the requests, the references that use the indexes in the data analysis and the methodological article that explains in detail how to calculate each one of them have been included. 
3) Discussion needs to discuss Brazil results in the context of regional inequalities in Latin America. 
Answer: after searching the literature, few studies carried out in Latin America dealing with a similar theme were found, however some comparisons have been made with the references we did find. They have been included in paragraphs one and seven of the discussion.
4) The quality and format of figures needs to be improved.
Answer: Figure 2 has been saved in WMF (created in STATA 15.1) format as requested by the journal and Figure 1 in JPG (created in excel).
