# Supplementary material for: Inequalities in care for the people with diabetes in Brazil: A nationwide study, 2019
Source: PLoS One. 2022 Jun 29;17(6):e0270027. doi: 10.1371/journal.pone.0270027 (PMC9242508; doi:10.1371/journal.pone.0270027)
Supplement: Attachment — Submitted filename: Response to Reviewers.docx [file pone.0270027.s002.docx]

February 28th, 2022

To the Journal Plos One

Subject: answer to editors and reviewers

Dear PLOS ONE Editor

We thank you for your careful review of the manuscript entitled “Inequalities in care for the people with diabetes in Brazil: a nationwide study, 2019”. We would like to point out that all requests have been met. Below are the details of each one and their respective answer.

**Major:**

1) Please describe in brief the health care system in Brazil, whether persons with diabetes have to pay for medical visits, examination and treatment, or these expenses are covered by the state? Who pay for glucose strips? Whether persons with higher education have health insurance from employer more frequent that those with only primary education? What is the proportion of out-of-pocket payments? How these differences could explain your findings? What is the role of community health centres, if any?

Answer: thanks, was added in paragraph 3 of the introduction explanation about the health system in Brazil.

2) You have mentioned that participants have been asked about administration of urine analysis, and indeed kidney disease is one of the major diabetes complications. You have provided (figure 2) very interesting data indicating that the differences in performing urine analysis were not so prominent between education groups compared to the differences in feet or eye examination, or HbA1c evaluation. How it is possible to explain?

Answer: in Brazil, the request for blood, urine and cholesterol tests is a protocol widely adopted in the management of NCDs, becouse they are simple and easily accessible tests for the patient. When we observe the examination of the eyes and feet by health professionals in the last year, the general prevalence decreases and the difference between the educational groups increases with higher frequencies of accomplishments in groups of higher socioeconomic status. There are studies in the country that show that carrying out these tests, especially in Primary Health Care, is low due to the unavailability of the necessary equipment, lack of training and knowledge of professionals and others. These points are highlighted in paragraphs 7, 8 and 9 of the discussion.

3) At line 222 please indicate how to interpret the "general PHC scores".

Answer: thanks, refers to the quality of care collected through the PCATool instrument.

4) It would be an advantage if you briefly describe how the people reported diabetes but not referred to a physician in a previous 12 months (and excluded from the analysis) differ from those reported diabetes and visited a physician.

Answer: the analysis was performed according to the availability of variables in the database. Our study investigated inequalities in the quality of care for people with diabetes through indicators of the work process of health professionals and for that, the sample considered was adults with DM who had consulted in the last three years. We do not have the separate information for consultation in the last year.

5) Please indicate what are the differences between High and higher education - that are among the categories you used in the analysis.

Answer: the education level was divided into five categories (no education; incomplete elementary school; complete elementary school / incomplete high school; complete high school / incomplete higher education and complete higher education). It was described in the methods.

6) The Table 2 will be much more easy to percept if you highlight in bold values with 95%CI higher than 1. Moreover, please use more clear column header and instead of putting "*PR: Prevalence Ratio" in the footnote provide the explanatory column header.

Answer: thanks, it was modified.

7) Please correct the tile for the Table 3 to make it more readable and avoid repetition of "concentration and".

Answer: thanks, done.

8) Please explain or comment in the "Discussion" why Slope index of inequality could be rather similar and in the same time concentration index could be rather different, for example considering the "Urine analysis", "Cholesterol or triglycerides" and "Feet examined in the preceding year".

Answer: the slope index of inequality (SII) and the concentration index (CIX) are different measures. The SII expresses absolute difference, in percentage points, between the prevalence of extreme education categories, using a logistic regression model. The CIX is based on a scale ranging from -100 to +100, with zero representing equal distribution across schooling categories, while positive values indicate that the distribution favors the most educated. The SII presents absolute inequality, while the CIX indicates relative inequality. This description was made in the penultimate paragraph of the methods.

9) Please indicate whether the National Health Survey data are accessible and open.

Answer: thanks, was added in the methods.

**Minor:**
1) Please revise the phrase "We performed all analyses using STATA® 15.0 statistical package, considering the sample design."

Answer: thanks, the phrases have been modified.

2) In the phrase "Advice on practicing physical activity and not drinking too much..." please specify whether drinking alcohol is considered or whatever.

Answer: thanks, the variable used was “recommendations for not drinking in excess”. It was described in the methods.

3) Please revise the phrases:
- "The greater the specificity of care, the greater the difference between schooling quintiles, with emphasis on the most educated."
- "The use of schooling in the present study can strengthen our findings..."
Answer: thanks, the phrases have been modified.

4) Please check consistency of English use in the table ("anos ou mais")

Answer: thanks, it was modified for “and over”.

5) Please use "95% CI" not "CI 95%"

Answer: thanks, it was modified.
