# Supplementary material for: Inequalities in care for the people with diabetes in Brazil: A nationwide study, 2019
Source: PLoS One. 2022 Jun 29;17(6):e0270027. doi: 10.1371/journal.pone.0270027 (PMC9242508; doi:10.1371/journal.pone.0270027)
Supplement: Attachment — Submitted filename: PlosOne11633.docx [file pone.0270027.s003.docx]

It is well written paper with 2019 data describing inequalities in DM care. I have only comments:

From the description of the Brazilian context on page 3 people could get the impression that SUS is the only health care system. According to my knowledge more than 25% of the population has an alternative health insurance paid privately or by the employer, that give them access to the huge private sector within the Brazilian health sector. I assume that access to private sector is much higher among well educated, and therefore explains a large part of the inequalities in care. This context needs to be described and discussed in the paper. It would have been interesting to look at the SII for different federal units, since that would tell us whether different state policies make a difference.

On page 11 line 271-275 it is indicated that education is used as proxy for economic level. Why? Education is often used as a measure of socioeconomic position in society. I agree that income or wealth could be very interesting measures but they might not be accessible to the authors. Those lines can be omitted.
